# The Relation between Persistent Post-Traumatic Headache and PTSD: Similarities and Possible Differences

**DOI:** 10.3390/ijerph17114024

**Published:** 2020-06-05

**Authors:** Martina Guglielmetti, Gianluca Serafini, Mario Amore, Paolo Martelletti

**Affiliations:** 1 Sant’Andrea Hospital, Regional Referral Headache Centre, 00181 Rome, Italy; martina.guglielmetti@uniroma1.it (M.G.); paolo.martelletti@uniroma1.it (P.M.); 2 Department of Clinical and Molecular Medicine, Sapienza University, 00181 Rome, Italy; 3 Department of Neuroscience, Rehabilitation, Ophtalmology, Genetics and Maternal Childhood Sciences, Psychiatry Unit, University of Genoa, 16132 Genoa, Italy; mario.amore@unige.it; 4 IRCCS Ospedale Policlinico San Martino, 16132 Genoa, Italy

**Keywords:** post-traumatic headache, migraine, persistent post-traumatic headache, neurovascular response to trauma, psychiatric comorbidity

## Abstract

Post-traumatic headache (PTH) may be considered a secondary headache, which is linked to severe disability and psychosocial impairment. Interestingly, nearly 30% of subjects with persistent post-traumatic headache (PPTH) also suffer from post-traumatic stress disorder (PTSD). Although existing studies demonstrated the existence of common pathophysiological characteristics in subjects with migraine and PPTH, the differences and similarities between these complex diseases are currently poorly understood and are yet to be comprehensively elucidated. Thus, the present review aimed to systematically investigate the nature of PPTH in the effort to better identify both the neurobiological and clinical aspects underlying this condition. Overall, the included studies reported that: (1) the predictors for persistent acute traumatic injury to the head were female gender, persistent symptoms related to mild post-traumatic brain injury (mTBI), PTSD, elevated inflammatory markers, prior mild traumatic brain injury, being injured while suffering from alcohol abuse; (2) static/dynamic functional connectivity differences, white matter tract abnormalities, and morphology changes were found between PPTH and migraine in brain regions involved in pain processing; and (3) clinical differences which were most prominent at early time points when they were linked to the increased risk of PPTH. Based on the selected reports, the relation between migraine and PPTH needs to be considered bidirectionally, but PTSD may play a critical role in this relation. The main implications of these findings, with a specific focus on PTSD, are discussed. Further longitudinal studies are needed to reveal the exact nature of this relation, as well as to clarify the distinct clinical characteristics of migraine, PPTH, and PTSD.

## 1. Introduction: Clinical Characteristics of Post-Traumatic Headache and Migraine, More than an Overlapping

Post-traumatic headache (PTH) is classified by the International Classification of Headache Disorders (ICHD) as a secondary headache occurring seven days after injury or trauma, recovering consciousness, and/or the ability to report pain [1]. The classification additionally subdivides PTH into an acute headache related to traumatic head injury, where the headache resolves within 3 months from onset, and a persistent headache related to traumatic head injury, where the headache persists beyond 12 weeks. Moreover, head trauma leading to PTH may be mild (usually associated with moderate, or severe mild traumatic brain injury (mTBI) [1].

PTH shows apparent phenotypes which are similar to migraine or tension-type headaches, and rarely cluster or cervicogenic headache phenotypes. PTH is usually linked to somatic symptoms (e.g., nausea, vomiting, phonophobia, and photophobia). Moreover, patients suffering from PTH and TBI also manifest cognitive and psychological symptoms, like anxiety and depression. Furthermore, around 30% of those exhibiting persistent PTH (PPTH) also reported post-traumatic stress disorder (PTSD), but this relation has been not systematically addressed [2,3].

Both migraine and PPTH need to be considered disabling conditions associated with relevant psychosocial impairment. Migraine affects about 14% of the global population (one billion worldwide), predominantly females in the age of increased productivity [4]. The Global Burden of Disease (2016) ranks it in third place in terms of frequency and second place in terms of disability (measured in years lived with disability, YLDs). From this point of view, migraine may be really considered a social illness. Migraine and PPTH patients have similar characteristics with PPTH patients who frequently present a migraine phenotype. Although some studies demonstrated the presence of white matter lesions in individuals with migraine [5], as well as in subjects with PPTH [6,7], the differences and similarities between these diseases are yet to be comprehensively elucidated.

The symptomatology of PPTH is very similar to that of migraine, as nausea, vertigo, difficulty concentrating, irritability, and fatigue characterize both diseases [8]. There are also other symptoms which are not well described in PTH, for instance, neck pain, premonitory symptoms (e.g., yawning, polyuria, food craving), aura symptoms such as visual disturbance, language difficulties, paresthesias, and mood/emotional lability. Aura is not common, although patients with PTH have migraine features; thus, it has been hypothesized that cortical spreading depression (CSD) is not a pathophysiological mechanism underlying PTH [9]. Some exacerbating migraine factors, such as stress, exercise, sleep, and hormonal changes, are less represented in PTH. Taking into account that mTBI may provoke some changes in cerebral spinal fluid (CSF), it may be useful to highlight whether orthostatism or clinostatism exacerbate the headache, and therefore changes in CSF pressure might have important consequences in PTH [9]. Moreover, breathing patterns may modify CSF flow [10,11] and breathing exercises may be considered as a useful treatment for PTH.

A headache after mTBI may be continuous and it could become discontinuous over time, but sometimes the headache might be continuous for a long period, while in others PTH takes place with delay, not immediately after trauma. These temporal characteristics allow the outlining of different phenotypes of headaches that underlie different mechanisms and need specific treatment [12].

The pathophysiological elements of acute mTBI are: axonal injury, cellular ionic fluxes, abnormal neurotransmitter release, cell swelling, an altered balance between cerebral metabolism and blood flow, and blood–brain barrier interruption [13].

Symptoms of mTBI (e.g., nausea, headache, consciousness loss, and head pressure) may be related to abnormally impaired neurotransmitter release, ion flux alterations, abnormal lactic acid release, enhanced blood flow in the central nervous system, increased glucose metabolism, energy deficit, and neuroinflammation [14].

## 2. Methods

In the effort to provide a comprehensive review concerning the similarities and differences between PPTH and migraine, we carried out a careful MEDLINE search to identify articles in English published between 2000 and 2019. Specifically, we searched for the following terms: “post-traumatic headache”, “chronic headache”, and “migraine symptoms and clinical characteristics”. The Medical Subjects Headings (MeSH) method was used in order to cross-reference specific terms with others regarding the field of search. Trials for inclusion were initially selected by the first reviewer (M. Guglielmetti), and later and independently a second reviewer (G. Serafini), who was blind to Dr. Guglielmetti, used the specified search strategy. When the identified abstract seemed to refer to a study potentially meeting the criteria for inclusion, the full-text article was downloaded to check the relationship with this review, according to the criteria for inclusion. Consultations with the senior author (P. Martelletti) were performed in order to resolve possible discrepancies between the two reviewers. In addition, we hand-searched the bibliographies from retrieved articles and published reviews. We also contacted study authors for further additional details/information concerning the selected studies.

While case reports and case series were considered for inclusion into this paper (for more details, see Table 1, Table 2 and Table 3), review articles and meta-analyses were not included, although we discuss these contributions within the main text where appropriate. Table 1, Table 2 and Table 3 were included for the aim of the present article and represent original material.

## 3. Results

### 3.1. Included Studies about PPTH

Overall, six studies investigated the nature of PPTH (see Table 1). One cohort study [15] showed the link between hospitalization related to head injuries of a mild degree, and new headaches associated with an aggravation of an existing headache. Three retrospective studies [16,17,18] reported that persistent headaches, beyond the diagnosis type, were linked to negative outcomes in terms of occupational functioning. They added that an increased severity in post-mTBI symptoms was present in middle-aged subjects relative to the oldest individuals, and they stressed the significant correlation between the type or severity of PTH and the rate of allodynia. Finally, according to two longitudinal prospective studies [12,19], traumatic brain injury was a strong predictor of headaches and psychiatric comorbidities, and headache symptoms were prevalent after mTBI, particularly throughout the first 12 months after being injured.

### 3.2. Included Studies about Migraine Neurobiological/Clinical Differential Predictors between PPTH and Migraine

Overall, 11 studies explored the existence of possible differential predictors of PPTH. Initially, six case–control studies [20,21,22,23,24,25] focused on the investigation of neurobiological/clinical differential predictors between PPTH and migraine. These studies reported: the existence of differences regarding static/dynamic functional connectivity among subjects with migraine and PPTH regarding brain regions related to pain processing; predominant clinical/neuropathological differences among PTH and non-PTH individuals at initial time points; abnormally bilaterally reduced cortical thickness in frontal areas and right hemisphere parietal regions in PPTH subjects; enhanced autonomic dysfunction in PPTH individuals; and abnormally decreased central nervous system (CSF) Tumor Necrosis Factor (TNF) alpha concentrations in most new daily persistent headache (NDPH) patients. Based on one cross-sectional cohort and another retrospective study without healthy controls as a comparing group [26,27], increased chronic reactive protein (CRP) concentrations at baseline in mTBI patients may independently predict the onset of persistent post-concussion symptoms (PPCSs), psychological impairments, cognitive dysfunctions, white matter tract alterations related to abnormalities in functional connectivity linked to abnormal pain modulation, and contribute to the onset/maintenance of chronic persistent headache (CPH). Moreover, in three longitudinal prospective studies [28,29,30], pathological imaging findings and female gender were reported as acute traumatic injury to the head (HAIH) predictors. Furthermore, previous mTBI, being injured while abusing alcohol, and acute HAIH were described as persistent HAIH predictors; the disrupted periaqueductal gray-default mode network (PAG-DMN) connectivity predicted PTH outcomes of mTBI individuals 12 weeks after injury; female sex, neck pain, two-week post-mTBI symptoms, and two-week post-traumatic stress were significant PPCS predictors (see Table 2).

### 3.3. Included Studies about Currently Available Treatments for PPTH

Overall, 10 studies examined the efficacy and tolerability of available treatments for PPTH (see Table 3). Two longitudinal prospective studies [31,32] found that IV metoclopramide 20 mg plus diphenhydramine 25 mg was effective and well tolerated in acute PTH subjects, while the interdisciplinary outpatient treatment of chronic headache subjects abnormally reduced the individual mean pain throughout the previous 7 days, together with actual pain severity (with a general maintenance of results at one-year follow-up). Moreover, according to four retrospective studies in this field [33,34,35], the multidisciplinary treatment approach was associated with reduced self-reported PTSD and persistent post-concussive symptoms (PPCSs), a relevant reduction in persistent post-concussive anxiety, dizziness, and headache was linked to identifying/correcting the visual misalignment with neutralizing prismatic lenses, and onabotulinum toxin A may represent a valid PPTH preventive strategy in subjects at risk for cognitive, metabolic, or behavioral adverse effects related to standard treatments. Furthermore, based on two cross-sectional studies [36,37], repetitive transcranial magnetic resonance (rTMS) influences headache severity, frequency, functional outcomes, symptoms of post-concussion, depression, and quality of life in PPTH subjects and patients with PPCSs, and rTMS was linked to a reduced severity in PPCSs and an enhanced activation of the dorso-lateral prefrontal cortex. Furthermore, two case series and one case report [38,39,40] reported that the behavioral treatment approach was an effective treatment for primary headache disorder and a good theoretical fit for treating PTH after mTBI. Electrical neuromodulation appears to be extremely beneficial for highly selected head-injured subjects with refractory PTH with disabling headaches, although comprehensive treatments and chronic refractory primary headaches may be successfully treated in the long-term with great auricular nerve (GAN).

### 3.4. A Focus on PTSD: Similarities and Differences with Persistent Post-Traumatic Headache

A high prevalence of chronic, persistent, daily headaches after mTBI, suggesting the existence of chronic migraine, which is closely linked to co-morbid PTSD, has been found in U.S. military forces [41]. The history of traumatic experiences and maltreatment is not directly related to predominant migraines in service members following deployment. Importantly, a pre-existing headache disorder may be enhanced by many factors such as head traumas, stress and major depression, PTSD, and environmental cues in the context of deployment [41]. Although PTH may be described as a secondary headache closely related to head trauma, a pre-existing migraine enhanced by mTBI in military populations after deployment may be similarly supposed. Usually, mTBI does occur in the combat-related context, leading to extreme physical and psychological consequences [42]. The associated injuries, as well as chronic pain disturbances, may enhance cognitive, somatic, or behavioral mTBI correlates. Combat-related mTBI is sometimes associated with an acute stress reaction, which is able to enhance psychiatric comorbidities and sleep disturbances, exacerbating behavioral and cognitive dysfunctions related to mTBI.

Based on existing studies [43,44,45], older age, female gender, problematic relations, comorbid conditions (e.g., prior psychiatric disorders, substance abuse/dependence, multiple injuries, mTBI history, and PTSD), and even psychosocial/situational factors are linked to a persistence throughout the course of the illness following mTBI.

Sports-related mTBI has been associated with depression or psychiatric conditions, attention deficit hyperactivity disorder, and sleep and learning disorders, together with chronic headache and migraine [46]. Interestingly, primary headache, sleep disturbances, stressors related to the psychosocial context, major depression and anxiety, substance abuse/dependence, in particular alcohol, physical injuries, or consequences of prior head injury have been reported both in military and civilian populations [47]. For instance, Rosenthal and Erickson [48] reported that in 270 army service members with PTH, 39% also met the PTSD diagnostic criteria. Similarly, a prevalence of 31% for PTSD in patients with chronic PTH after minor head trauma has been reported by Kjeldgarrd et al. [2], while Peterlin and colleagues [49] reported predominant PTSD symptoms in samples of migraine. Post-concussive disorders, regardless of the presence or absence of mTBI, seem to be predicted by pre-injury depressive and anxiety disorders and chronic pain, according to the study of Meares and colleagues [50], who stressed the nonspecific nature of symptoms after mTBI, which are conversely reported, based on their point of view, in the chronic pain population. A predominance of chronic pain with unclear origin after traumatic brain injury has been found in 51% of civilian and 43% of military cohorts, suggesting the importance of “early screening” and pain treatment [51]. Furthermore, further associated factors of persistent post-mTBI syndromes were cumulative stressors and coping strategies [52,53]. After investigating a sample suffering from mild to moderate TBI, McCauley and colleagues [54] found the presence of reduced social support and abnormally enhanced self-reported depressive symptoms four weeks after mTBI, together with concurrent comorbid PTSD and major depression.

In addition, headaches linked to trauma, aura, opioid abuse/dependence, and comorbid psychopathology were found to be associated with negative outcomes and decreased rates of return to duty based on a recent retrospective report, which investigated a sample of service members who were recruited from war zones due to headache in the context of deployment [55]. Unfortunately, the consequences of the early detection and treatment of comorbid disabling conditions, that were reported to impact significantly on individual wellbeing following mTBI in civilian and military populations, are poorly understood.

## 4. Discussion

### 4.1. Summary of the Main Findings about the Nature of PPTH

All the considered [56] studies about the nature of PPTH concluded that: (1) being exposed to head injury enhanced the risk of headache onset and aggravation; (2) the presence of continuous headache was strongly linked to negative occupational outcomes; (3) the careful identification of symptoms in the initial phase of headache may help clinicians to understand the illness prognosis and avoid chronicity and enhanced disability; (4) subjects with PPTH were most likely to present continuous pain (see Table 1).

These studies define PTH as a specific diagnostic entity characterized by a common onset after head traumatic experiences. In the ICHD, PTH is identified as either acute or chronic, occurring after mild or severe traumas, with considerable variability regarding the mechanisms of action, clinical characteristics, and longitudinal illness course. Moreover, the nature of the trauma, for instance, motor vehicle accidents and sport or blast injuries, and the involvement of the neck, may result in distinct phenotypes with different illness trajectories [9]. In a study based on soldiers, Wilk et al. reported that in a sample of soldiers suffering from mild TBI associated with a loss of consciousness, when compared to non-blast injuries, blast injuries were more likely to occur related to persistent headache [57]. Repeated head/neck trauma could be responsible for multiple PTH phenotypes relative to an isolated trauma. From this point of view, clarifying the mechanisms underlying trauma and whether trauma may be considered a unique or recurrent manifestation may clinically help to understand the different types of headache [9].

Most PTH subjects present features of migraine but these characteristics may be considered variable. Pain related to PTH is usually unilateral, pulsating in quality, severe, and it worsen with physical activity. However, the intensity of PTH and the frequency of attacks generally varies in different treatments. Other reports report that PTH in TBIs of all severities is mostly classified as migraine or probable migraine, and hypothesize common pathophysiological mechanisms for these two diseases, with PTH that may respond to migraine-specific therapies (e.g., triptans) [12]. PTH patients usually reported a positive history of traumatic experiences and maltreatment that may be, however, not temporally related to the occurrence of migraine. Importantly, a prior history of trauma and comorbid psychopathology were strongly linked to negative outcomes in the PTH patient population.

### 4.2. Summary of the Main Findings about Neurobiological/Clinical Differential Predictors between PPTH and Migraine

All the considered [29] studies concluded that: (1) the predictors for persistent acute traumatic injury to the head were female gender, persistent symptoms after mTBI, PTSD, elevated inflammatory markers, prior mild TBI, being injured due to alcohol abuse/dependence; (2) based on functional and structural imaging, static/dynamic functional connectivity differences, white matter tract abnormalities, and brain morphology changes were found between PTH and migraine for regions involved in pain processing; (3) differences between PTH and non-PTH patients were predominant in the initial illness phases when they were linked to an increased risk of PTH.

Female gender is, in our opinion, a predictor for the development of persistent post-traumatic headache. However, some studies reported that females were more likely to suffer from acute traumatic injuries to the head.

Importantly, the history of prior migraine episodes has been reported to enhance the risk of post-traumatic migraine [29]. It has been hypothesized that the neurovascular trauma response can be enhanced by migraine mechanisms, resulting in persistent and more disabling symptoms after mTBI. Alternatively, mTBI may be able to activate, in individuals with a specific migraine predisposition, a preexisting headache (and perhaps migraine) mechanisms [58].

According to a comprehensive perspective, mTBI and other brain-related traumas may cause persistent headache and migraine in individuals with genetic vulnerability. Migraine has strong genetic roots with some genetic polymorphism specifically related to type 1 familial hemiplegic migraine (FHM1) (CACNA1A gene encoding P/Q type calcium channel) and type 2 familial hemiplegic migraine (FHM2) (ATP1A2 gene encoding Na/K ATPase), associated with differential phenotypes in response to minor head trauma [59]. A mild head trauma might provoke hemiplegic migraine attacks. Previous studies showed how severe migraine symptoms may be induced even after minor traumas by P/Q type calcium channel or Na/K pump genetic alterations, hypothesizing the existence of similar mechanisms underlying PTH susceptibility, even among individuals without FHM. Therefore, clinicians may consider both the P/Q type calcium channel and Na1/K1 ATPase as possible therapeutic targets in PTH subjects [9]. It is also important to consider the psychological consequences related to mTBI and trauma-related headache. To this specific regard, anxiety, depression, and PTSD are collectively associated with headache and severe/frequent headaches are linked to preexisting mood and anxiety disorders in a dose–response manner [60].

Given this background, head injuries may activate the same mechanisms underlying migraine or migraine tension-type headache only in a specific (more vulnerable) subgroup of patients. The initial headache symptoms might develop to be persistent due to psychiatric comorbidity and perceived pain, further exacerbating the psychosocial impairment of the initial mTBI.

### 4.3. Summary of the Main Findings about Currently Available Treatments for PPTH

All the considered [36] studies concerning currently available treatments for PPTH concluded that: (1) specific neuromodulation techniques (e.g., rTMS and electrical neuromodulation in the short-term period, and the great auricular nerve as a long-term treatment) may influence headache severity, frequency, functional outcomes, post-mTBI symptoms, psychiatric symptoms, and quality of life; (2) behavioral treatment and interdisciplinary outpatient treatment approaches may be effective nonpharmacological treatments for headache disorders; (3) specific pharmacological strategies, such as metoclopramide plus diphenhydramine and botulinum toxin A, may be able to restore most subjects to a normal psychosocial functioning and eliminate symptoms related to mTBI.

According to the current literature, treatments have similar effects, while fremanezumab (CGRP MoAb) seems to be a promising tool for both headache forms [61]. There are many therapeutic targets that could be explored in clinical trials, starting from the current available treatments, like exercise, non-steroidal anti-inflammatory drugs, antagonists of substance P receptors (NK1) or glutamate receptors, adenosine receptor antagonists and agonists, and hormonal treatments [9]. Glutamate deserves a further mention because it may be considered the main excitatory neurotransmitter involved in migraine pathophysiology and there are robust preclinical studies showing that glutamate release or specific glutamate receptor alterations may be induced by TBI [62]. High levels of glutamate have been observed in the serum and in the CSF, both ictally and interictally, in migraine. Early treatment targeting glutamate receptors with magnesium supplementation may be an important option in PTH and in migraine [9]. Recent studies showed that migraine may be associated with a calcitonin gene related peptide (CGRP), and specific treatments targeting CGRP, as well as CGRP receptors, like the monoclonal antibodies, have proved their efficacy in clinical trials. Animal models showed how CGRP is released after TBI and influences migraine-related behaviors like hyperalgesia and photophobia. Therefore, these two findings may suggest that CGRP might also play an important role in PTH [63,64].

### 4.4. Main Caveats

The present review needs to be considered according to the following limitations/shortcomings. First, most reports did not investigate the time of headache onset or use self-report instruments to explore the presence and severity of PTH. In addition, the preliminary/exploratory nature of some studies selected for this review manuscript, as well as the inclusion of case reports or case series, could not have allowed the generalization of the most relevant study findings. The absence of a study control group in most of the cohort included studies, the extremely small sample sizes, together with the selection of some studies using a cross-sectional or retrospective design, need to be addressed as further major caveats that do not allow the drawing of conclusions. Furthermore, we included some clinical cohort epidemiological reports which are known for having low sample sizes and a focus on individual outcomes to support our findings, and studies that had adopted retrospective designs, thus the findings may have been hampered by recall bias. In addition, the selection of study cohorts was highly focused on studies done in military populations suffering from battlefield-related trauma. Although soldiers need to be considered the predominant population when we refer to PTSD prevalence, this could represent a bias.

Moreover, patients with PPTH represent a very select group of patients that may present migraine-like headaches; unfortunately, there are no studies, to our knowledge, that have been carried out in order to primarily identify the similarities and differences between PPTH and migraine. Thus, all the conclusions about the nature of PPTH and migraine have been interpreted indirectly and according to the most relevant selected studies about the main topic. Furthermore, information about headache severity, as well as the presence of psychiatric comorbidities, was not available in most studies, and this may have biased the main study results. Finally, the link between post-traumatic headache and migraine certainly will not be solved in a review article that may foster further controlled studies in this respect.

## 5. Conclusions

Based on the most relevant studies about the main investigated topic, the relation between migraine and PPTH need to be considered bidirectionally. It has been hypothesized that the neurovascular trauma response can be enhanced by migraine mechanisms, resulting in persistent and more disabling symptoms after mTBI. However, mTBI may alternatively be able to activate the same neurobiological mechanisms underlying tension-type headache or migraine only in a specific subgroup of patients, raising the possible existence of a genetic vulnerability. In line with this assumption, patients suffering from migraine report a persistent long-term exacerbation of their migraine/headache after head trauma. In both cases, the presence of PTSD may play a relevant role in disability and psychosocial impairment related to PPTH. The current literature upon this topic is growing rapidly, showing a spreading interest in this area in terms of diagnostic tool applications, differential diagnosis among headaches, and epidemiological data from emergency and psychiatric departments, which are the ideal frame for unbiased studies on these patients, in order to have more valid data in the future that will help us understand if this research path is worth pursuing [65,66,67,68,69,70,71]. Importantly, we have also to carefully address the link between emotional turmoil, which is closely linked to suicidal behavior, in PTH patients. Understanding this is crucial to attenuate stigma [72], improve illness outcome, and provide a comprehensive and appropriate treatment [71,73,74,75,76]. The relevance to the collection of reliable information in the evaluation of suicide risk cannot be ignored by physicians when approaching patients with PTH [77,78]. A fascinating journey is only in its initial phases and further longitudinal studies are needed to reveal the exact nature of the relations between PTSD, migraine, and PPTH, although the enthusiasm of these times seems to be premature.

## Figures and Tables

**Table 1:** Most relevant clinical studies about persistent post-traumatic headache (PTH).

Author(s)	Sample	Study Design	Main Findings	Limitations	Conclusions
Nordhaug et al., 2018 [15]	Two hundred and ninety-four inpatients according to a 11-year time period (exposed) and 25,662 subjects who were not hospitalized in the same time period (unexposed).	Cohort study.	Both headache onset and aggravation may be enhanced by being exposed to head injury.	The time of headache onset has been not determined.	When compared to the general population, inpatients with mild head injury are at elevated risk of headache onset and aggravation.
Finkel et al., 2017 [16]	Ninety-five assessed during a 12-month time period.	Retrospective observational study.	Negative occupational outcomes are linked to persistent headache, beyond the diagnosis type. Headache diagnosis type was not linked to soldiers’ separations from service beyond headache duration.	Military injuries may be considered not specific in other populations. The generalization of the main findings is influenced by the small sample size and single examiner.	Subjects with PPTH are at increased risk of persistent pain. The most frequent primary diagnosis type is migraine. Negative occupational outcomes were linked to persistent headache.
Hu et al., 2017 [17]	One hundred and sixty-seven outpatients after mTBI.	Retrospective analysis of prospectively collected data.	Relative to patients over 66 years of age, that are more likely to report mTBI between 6 a.m. to 12 p.m. (69%), middle-aged patients (36–55 years) reported a higher severity of specific post-mTBI symptoms.	The study used self-report instruments. The study did not evaluate prior mTBI episodes or psychiatric disorders following mTBI. Subjects were recruited in a tertiary care center and cannot be generalized to all mTBI populations.	Relative to the oldest patients, middle-aged subjects reported more severe symptoms after mTBI. A link between age and severity of mTBI symptoms has been identified.
Markus et al., 2016 [18]	Seventy-four patients, of which 60 with mild and 14 with moderate/severe TBI.	Retrospective review of the computerized files.	The recruited subjects reported a lower rate of migraine-like headache and a higher rate (in particular in males) of allodynia. PTH type/severity and rate of allodynia were not correlated.	The study includes a very selected sample of individuals. It could not be determined how many of the children with TBI continued to manifest headache or other headache correlates.	Children with mild/severe TBI more frequently manifest migraine-like and tension-like headaches. Children with PTH frequently had allodynia (common mechanisms related to central sensitization in PTH and primary migraine have been supposed). PTH may be treated using active treatments for migraine.
Jaramillo et al., 2016 [19]	Iraq and Afghanistan war veterans (38,426) who were treated at baseline (in 2008) and in the following years (2009, 2010, 2011).	Longitudinal retrospective cohort study.	TBI alone was a strong predictor of headache and psychiatric comorbidities among subjects with TBI. Importantly, insomnia, tinnitus, and vertigo predicted headache persistence only in individuals with baseline headache.	Misclassification due to ICD-9-CM code use may be the first caveat. Information about condition severity was not available. Findings may be influenced by the restriction of the analyses to subjects with Veterans Health Administration care in each year of evaluation (due to the inclusion of only the most severe individuals). Not all subjects with depression, PTSD or TBI were included in the study.	The careful identification of symptoms in the initial phases of headaches may help clinicians to understand the illness outcome.
Lucas et al., 2014 [12]	Two hundred and twelve inpatients within a seven-day period after mTBI who were assessed by telephone 12, 24, and 48 weeks after injury (according to ICHD-2 criteria).	Prospective study.	Overall, headaches pre-injury was found in 18%, headache onset or aggravation related to the existence of pre-injury immediately in 54%, 62% at three months, at 24 weeks in 69%, and at 48 weeks in 58% of subjects. Cumulative incidence was 91% after 48 weeks. Migraine was reported in nearly 49% and tension-type headaches in 40% of all headaches.	Only self-report instruments were used. Additionally, the investigation of only one headache type at each time period represents a further caveat.	Throughout the first 12-month period after injury headache, mTBI is very frequent and persistent. Chronicity and disability may be prevented with assertive/early treatment.

**Note:** mTBI = mild traumatic brain injury; post-traumatic = PT; persistent post-concussion symptoms = PPTH; post-traumatic stress disorder = PTSD.

**Table 2:** Most relevant studies focusing on neurobiological/clinical differential predictors between persistent post-traumatic headache and migraine.

Author(s)	Sample	Study Design	Type of Intervention/Procedure	Main Findings	Limitations	Conclusions
Dumkrieger et al., 2019 [20]	Thirty-three patients with migraine, 44 with persistent post-traumatic headache, 36 HC.	Case–control study.	Fifty-nine a priori brain regions of interest related to pain processing were selected. The connectivity patterns of these regions were investigated statically/dynamically.	Migraine and PPTH patients may be distinguished in terms of different (static and dynamic) functional connectivity related to specific pain- and visual-processing brain regions.	Functional connectivity results presumably due to PPTH vs. findings related to underlying mTBI may be not dissected. No information about the rate of participants with migraine at the time of imaging are available. Half of migraine and PPTH patients were using preventive drugs.	Functional imaging showed functional connectivity differences between migraine and PPTH in specific regions of interest (related to pain processing), postulating distinctive pathophysiology linked to migraine vs. PPTH.
Burrowes et al., 2019 [21]	Fifty mTBI patients (of which 31 non-PTH; 19 PTH) and 21 HC.	Cross-sectional study.	MRI scans were carried out after 10 days, 4, 24, and 72 weeks post injury. A specific headache questionnaire was used to assess PTH during visit four after TBI.	Abnormally reduced GMV in the right anterior-parietal and left temporal operculum were found in PTH individuals reported. Reduced GMV in the left thalamus were reported in non-PTH subjects compared to HC as well. Reduced GMV in left temporal operculum, superior frontal gyrus, temporal parietal junction, right middle frontal gyrus, superior frontal gyrus, and anterior parietal cortex were finally reported in PTH patients.	Patient headache status was not assessed before injury. In addition, there is a possible recall and selection bias linked to the administration of the headache questionnaire at visit four.	Initial differences linked to an increased risk of PTH were predominant between PTH and non-PTH.
Chong et al., 2019 [22]	Forty-nine PPTH subjects due to mTBI, 41 with migraine, and 41 HC.	Cross-sectional study.	Eighteen fiber tracts were reconstructed for 131 subjects.	There were relevant differences between migraine and PPTH (in terms of mean or radial diffusivity) in the cingulum, inferior longitudinal fasciculi, bilateral anterior thalamic radiations, the right superior longitudinal fasciculi-parietal portion, left corticospinal tract, and uncinate fasciculi. In migraine individuals, headache frequency and forceps major mean diffusivity were correlated, while a correlation between headache frequency and cingulum angular bundle mean and radial diffusivity was found in PPTH.	Only episodic and chronic subjects with migraine were considered. The statistical model was not controlled for eventual mood instability. Medication overuse individuals were not assessed in both PPTH and migraine subjects. Results may be influenced by the history of specific psychiatric disorders.	Results hypothesized specific differences in the neuropathological mechanisms related to both migraine and PPTH.
Chong et al., 2018 [23]	Thirty-three patients with PPTH and 33 HC.	Cross-sectional cohort study.	Brain MRI (3 tesla scanner) was used for subjects and HC.	Lower cortical thickness was found in PPTH patients, relative to HC, in the caudal middle frontal, left, right superior frontal, and precentral cortex, together with lower cortical thickness in the right superior and inferior parietal, right supramarginal, and right precuneus region. A negative correlation between right and left superior frontal thickness with headache frequency, according to lower cortical thickness, was reported in PPTH participants.	The study does not permit to disentangle the effect that mood dysfunctions may have had on altering cortical thickness patterns and brain changes related to concussion vs. those associated with headaches in PPTH patients. Finally, the study did not collect information on medication intake, history of smoking, and metabolic indices.	Lower cortical thickness in the right hemisphere parietal regions and bilateral frontal regions were found in PPTH patients. The study hypothesized that brain morphology changes in the superior frontal regions were modified by headache frequency in PPTH patients.
Howard et al., 2018 [24]	Fifty-six PPTH patients, 30 migraine subjects, and 36 HC.	Cross-sectional cohort study.	Participants were assessed with COMPASS-31 questionnaire.	PPTH and migraine individuals manifested higher COMPASS-31 mean total scores than HC. Among subjects with PPTH, total lifetime TBI was positively linked to COMPASS-31 total scores, years spent with headache with vasomotor subdomain, and headache frequency with vasomotor subdomain.	The range of possible autonomic symptoms was not assessed. Moreover, subjects with PPTH reported fewer headaches, lower years spent with headaches, and a higher rate of males. Subject recruitment was obtained with a convenience sampling. Findings were not corrected for multiple calculations.	PPTH subjects exhibited a higher rate of autonomic symptoms.
Rozen and Swidan, 2007 [25]	Thirty-eight patients (20 with NDPH and 16 HC patients with CM, and two with PTH).	Cross-sectional cohort study.	CSF and serum TNF alpha levels were detected.	Ninety-five percent of NDPH patients and 100% of CM and PTH participants reported abnormally elevated CSF TNF alpha levels. Most of the individuals exhibited normal serum TNF alpha levels.	The study results need to be considered as preliminary. The relatively small sample size does not permit to generalize the most relevant results.	Approximately all NDPH patients reported an abnormal increase in CSF TNF α concentrations, suggesting a specific role for TNF α in the pathophysiology of NDPH.
Leung et al., 2018 [26]	Twelve mTBI veterans.	Cross-sectional study.	DTI data were acquired.	The mTBI cohort showed white matter abnormalities associated with pain affective and modulatory functions in the right anterior thalamic radiation and left superior longitudinal fasciculus. Moreover, the mTBI cohort exhibited a decrease in axial/radial diffusivity at the superior longitudinal fasciculus cluster.	Controls were composed of healthy subjects and not by mTBI subjects without headache. In addition, the sample size of this study needs to be considered as quite small.	CPH seems to be related to specific white matter tract abnormalities linked to functional connectivity dysfunction in pain modulation.
Su et al., 2014 [27]	Two hundred and thirteen consecutive patients with mTBI.	Retrospective study.	Plasma CRP concentrations were measured at baseline, 4, 8, and 12 weeks after initial TBI.	A significant increase in persistent PPCSs (OR = 2.719), persistent psychological distress (OR = 1.535), and persistent cognitive dysfunctions (OR = 1.687) were linked to enhanced baseline CRP levels. Persistent physiological problems (OR = 1.330) were linked to elevated CRP levels.	The study design, and the relatively small sample size, do not allow to generalize the most relevant results to other populations and countries. Findings may have been interpreted with subjectivity.	Abnormally elevated CRP concentrations at baseline may independently predict PPCSs, psychological distress, and cognitive dysfunctions in mTBI subjects.
Nordhaug et al., 2019 [28]	Three hundred and seventy-eight subjects first exposed to mTBI, 82 trauma (exposed to minor orthopedic injuries) controls, and 83 community (exposed to all injury types) controls.	Population-based, controlled, prospective cohort study.	Questionnaires were used at baseline, 12, and 48 weeks after injury.	Individuals with HAIH over the course of the first 12 weeks after injury may significantly improve before 48 weeks after injury. Headache aggravation may be predicted by female sex, CT/MRI results, history of positive mTBI, and being injured when affected by alcohol abuse/dependence.	Recall bias needs to be considered a major shortcoming (participants are included after their mTBI).	Headache aggravation is more frequently correlated to head injury after 12 weeks following MTBI. Specific factors predicted headache aggravation.
Niu et al., 2019 [29]	Seventy patients with mTBI and 46 HC.	Longitudinal follow-up study.	Neuropsychological measurements and MRI scans were carried out within 7 days post injury, with 80% of subjects followed for 12 weeks.	mTBI + APTH patients presented reduced PAG-seeded FC within the DMN compared with HC. The initial FC strength between the PAG-right precuneus and the PAG-right inferior parietal lobule became the important predictor to identify patients with mTBI developing persistent PTH 3 months post injury.	The study findings need to be considered as preliminary. The relatively small sample size does not permit to generalize these exploratory results.	The disrupted PAG-DMN connectivity was predictive of PTH outcomes of subjects with mTBI after 12 weeks of follow-up.
Cnossen et al., 2018 [30]	Five hundred and ninety-one participants.	Prospective study.	The Head Injury Severity Checklist was used to evaluate PPCSs at six months after injury.	PPCSs were identified in 241 (41%) patients. Female sex (OR = 1.48, neck pain (OR = 2.58), one-month symptoms after concussion (OR = 4.89) and one-month PTSD (OR = 2.98) were identified as possible predictors in the identified model.	Many patients were lost at the follow-up. Selection bias, which could have influenced the significance of predictors, cannot be excluded as well. The outcome measurement differed from the outcome measurement used in both development studies. Finally, symptoms were only included if there was evidence of deterioration.	The model, including female sex, complaints at the emergency department, two-week PPCSs, and post-traumatic stress as predictors, performed reasonably and may therefore be potentially valuable for clinical practice.

**Note:** Acute post-traumatic headache = APTH; acute traumatic injury to the head = HAIH; chronic migraine = CM; chronic persistent headache = CPH; C-reactive protein = CRP; default mode network = DMN; dorsolateral prefrontal cortex = DLPC; Diffusion tensor imaging = DTI; Functional connectivity = FC; grey matter volume = GMV; healthy controls = HC; mild traumatic brain injury = mTBI; new daily persistent headache = NDPH; periaqueductal gray-default mode network = PAG-DMN persistent post-traumatic headache = PPTH; persistent post-concussion symptoms = PPCSs; post traumatic headache = PTH.

**Table 3:** Most relevant clinical studies reporting the importance of currently available treatments in persistent post-traumatic headache.

Author(s)	Sample	Study Design	Type of Intervention/Procedure	Main Findings	Limitations	Conclusions
Friedman et al., 2018 [31]	Patients with moderate/severe headache were recruited at ED and contacted for re-evaluation by telephone 2 and 7 days later.	Prospective open-label study.	Metoclopramide 20 mg plus diphenhydramine 25 mg was used to treat participants.	Acute PTH has been successfully treated using IV metoclopramide 20 mg plus diphenhydramine 25 mg. Subjects have been restored to normal functioning and concussion symptoms have been deleted.	The open-label study design may result in an overestimation of treatments efficacy. The relatively small sample size does not allow to appropriately identify the outcomes.	Subjects with acute PTH may be successfully treated using IV metoclopramide 20 mg plus diphenhydramine 25 mg. Importantly, 33% of participants manifested headache relapse after discharge and 25% had persistent headaches 7 days later.
Krause et al., 2017 [32]	Three hundred and seventy-nine outpatients admitted between 2008 and 2011.	Prospective study.	A 21-day interdisciplinary treatment program.	A relevant reduction in terms of headache severity, psychological distress, and disability after the program was reported. Improvements in functioning were maintained at 48 weeks. HIT-6 scores improved further after discharge.	No control group was included. The selection if the study sample may be not focused on the program’s aims. Only 40% of the baseline sample were longitudinally followed. Findings may be biased by the reduced survey return rate.	The present results supported the efficacy of the used 21-day interdisciplinary treatment program. Assessments at a 12 month follow-up confirmed the baseline improvements.
Janak et al., 2017 [33]	Two hundred and fifty-seven active-duty subjects with mTBI who performed a multidisciplinary outpatient treatment (2008–2013).	Retrospective study.	Pre- and post-treatment changes in both PTSD and PPCS subjects were measured. Cognitive rehabilitation, vestibular interventions, headache management, and integrated behavioral healthcare were all included in the comprehensive investigation.	Military subjects with a self-reported mTBI who completed multidisciplinary treatment reported a reduction in both PTSD and PPCSs.	This was an exploratory observational study that used retrospective data originally collected for clinical purposes. A large proportion of participants were excluded due to missing PT assessment data.	The multidisciplinary treatment approach was associated with reduced self-reported PTSD and PPCSs.
Rosner et al., 2016 [34]	Thirty-eight participants having symptoms after concussion.	Retrospective study.	Assessments were carried out before and after prism application. A further evaluation of symptoms related to heterophoria was performed using a 10 cm visual analogue scale at the end of study treatment.	Persistent symptoms of anxiety, dizziness, and headache after concussion were attenuated with multiple metrics identifying/correcting the visual misalignment with neutralizing prismatic lenses. A significant reduction in headache, dizziness, anxiety, and overall subjective symptoms was reported.	Patients diagnoses were obtained using only history, physical findings, and the prism lenses positive response. Amounts of vertical misalignment cannot be measured using a single device or test.	Dizziness and anxiety in participants with persistent symptoms after concussion may be significantly treated with neutralizing prismatic lenses.
Yerry et al., 2015 [35]	Sixty-four chronic PT headache and 47 chronic migraine patients between August 2008 and August 2012.	Real-time retrospective consecutive case series.	FDA-approved injections (site/fixed dose), combined with FTP and cervical dystonia, were used. Electronic medical records (derived by the Armed Forces Health Longitudinal Technology Application) were used to collect socio-demographic and treatment features, as well as clinical/occupational outcomes.	OBA may be effective for PPTH in a population at risk for cognitive, metabolic, or behavioral adverse effects.	The small number of highly selected patients within the study sample does not permit to generalize the most relevant results.	OBA may be effective in the analyzed sample with headaches correlated to concussion.
Stilling et al., 2019 [36]	Twenty patients with PPTH and PPCSs.	Double-blind, sham-controlled, concealed allocation, randomized clinical trial.	A random number generator was used to randomize participants (parallel assignment).	rTMS influences headache severity, frequency, functional outcomes, symptoms after concussion, depression, and quality of life in subjects with PPTH and PPCSs.	The small sample size may have been underpowered to identify significant changes in our outcome measures. The rTMS intensity was on the lower end of previously reported studies.	This study showed full participant adherence with no dropouts, a 100% questionnaire response rate, no serious adverse effects, and an efficacious blinding method.
Koski et al., 2015 [37]	Fifteen adult subjects with persistent PPCSs 24 weeks or more after injury.	Pilot cross-sectional study.	Twenty sessions of rTMS were conducted over four weeks.	Post-acute TBI symptoms were significantly attenuated by rTMS.	No control group has been included; this does not permit to generalize the most relevant results.	rTMS is effective (as sustained by the attenuation in PPCS severity increase in task-related DLPFC activation) in a sample with acute post-TBI symptoms.
Silverberg, 2019 [38]	Four participants with PTH (related to mTBI) recruited 36–76 weeks following injury.	Case series.	An eight-session manualized procedure with a registered psychologist was performed. Participants carried out a daily headache diary and pre- and post-treatment evaluations using questionnaires before, during, and after treatment.	A variability in terms of improvement was reported.	The generalization of the main findings is influenced by the study design and the relatively small sample.	The behavioral treatment approach is an effective nonpharmacological therapy for primary headache disorder and is a good theoretical fit for treating PT headaches after mTBI.
Elahi and Reddy, 2014a [39]	A 40-year-old man with persistent daily headaches (recruited after motor vehicle accident without prior headache episodes).	Case report.	Implantation of the SCS electrode was performed after the subject showed a 90% improvement in pain during the 7 days of a percutaneous SCS trial.	This participant reported at least 90% pain attenuation and improvement in terms of quality of life during the 7 days of the high cervical dorsal column electrical SCS trial. He underwent a permanent implantation of high cervical dorsal column electrical nerve stimulation and reported a similar pain reduction along with 100% satisfaction rate at the 12-month re-evaluation.	The study design does not permit to generalize the main results.	Electrical neuromodulation appears to be extremely beneficial for highly selected head-injured subjects suffering from PPTH.
Elahi and Reddy, 2014b [40]	A 57-year-old male having chronic, intractable PTHs.	Case report.	A long-term electrical neuromodulation of the C2–C3 branches within the GAN distribution has been carried out.	This participant reported a significant headache attenuation following 6 months of permanent peripheral neurostimulator implantation.	The study design does not permit to generalize the main findings.	The GAN appears as an effective long-term treatment in subjects with chronic primary headaches.

**Note:** Emergency department = ED; intravenous = IV; great auricular nerve = GAN; left dorsolateral prefrontal cortex = DLPFC; mild traumatic brain injury = mTBI; persistent PTH = PPTH; onabotulinum toxin A = OBA; persistent post-concussive symptoms = PPCSs; post-traumatic = PT; post-traumatic stress disorder = PTSD; repetitive transcranial magnetic stimulation = rTMS; spinal cord stimulation = SCS.
